# Revised Short Screening Version of the Profile of Mood States (POMS) From the German General Population

**DOI:** 10.3389/fpsyg.2021.631668

**Published:** 2021-05-31

**Authors:** Katja Petrowski, Cornelia Albani, Markus Zenger, Elmar Brähler, Bjarne Schmalbach

**Affiliations:** ^1^Medical Psychology and Medical Sociology, University Medical Center of the Johannes Gutenberg-University, Mainz, Germany; ^2^Department of Internal Medicine III, University Medical Center Carl Gustav Carus at the University of Dresden, Dresden, Germany; ^3^Schussental Clinic, Clinic for Psychosomatic Medicine and Psychotherapy, Aulendorf, Germany; ^4^Faculty of Applied Human Studies, University of Applied Sciences Magdeburg and Stendal, Stendal, Germany; ^5^Integrated Research and Treatment Center (IFB) AdiposityDiseases - Behavioral Medicine, Psychosomatic Medicine and Psychotherapy, University of Leipzig Medical Center, Leipzig, Germany; ^6^Department of Psychosomatic Medicine and Psychotherapy, University Medical Center of the Johannes Gutenberg-University, Mainz, Germany

**Keywords:** mood, assessment, scale development, screening instrument, factor analysis

## Abstract

The present study was conducted with the aim of constructing and validating a short form of the Profile of Mood States (POMS). The POMS is a widely-applied measure for the assessment of an individual's mood. Thus, it is of great relevance for many research questions in clinical and social psychology. To develop the short scale, we first examined psychometric properties and found the optimal 16-item solution among all valid combinations of the full POMS in an exploratory subsample (*n* = 1,029) of our complete representative sample of the German general population. We then validated this model in a confirmatory subsample (*n* = 977). Additionally, we examined its invariance across age groups and sex, as well as its reliability. Our results indicate that the POMS-16 is a valid and reliable measure of mood states with minimal losses compared to the 35-item version. Particularly where brevity and an economical assessment is desired, the POMS-16 should be considered.

## Introduction

The questionnaire “Profile of Mood States” (POMS; McNair et al., 1992) is a widely used questionnaire in the clinical field when examining psychotherapeutic, psychological and somatic questions, but also in pharmacological, occupational, and sports medicine studies to record the state of mind. In a wide variety of medical subjects, additionally, the POMS is used to examine groups of patients, e.g., patients with cataract (Pesudovs et al., 2003), with sleep apnea syndrome (Bardwell et al., 2003), with epilepsy (von Steinbüchel et al., 1994; Szaflarski et al., 2003), or with heart surgery (Gross, 1991). Especially in the (psycho) oncological area, it is used to measure the general stress on patients (Dilorenzo et al., 1999), but also the quality of life (e.g., Baker et al., 2002) and to evaluate the effects of interventions (e.g., Classen et al., 2001; Hosaka et al., 2001; Grulke et al., 2004). These application areas are consistent with the seven areas of application of the POMS described in the manual by the authors McNair et al. (1992): psychotherapeutic and pharmacological studies, cancer and addiction research, research on emotions as well as in sports psychology.

The American original version (McNair et al., 1971, 1992) contains 65 items that are answered on a 5-point scale (“0 = not at all” to “4 = very strong”) for the period of the “past week, including today” which belong to following scales: Depression, Tension-Anxiety, Anger-Hostility, Fatigue-Inertia, Vigor-Activity, and Confusion-Bewilderment. The Total Mood Disturbance Scale is formed by subtracting the (positive) value of the Vigor scale from the sum of the remaining five scales. For the six scales, the coefficients for the internal consistencies range between 0.90 and 0.94 and the retest reliabilities (median 20 days apart) between 0.65 and 0.74 for samples of psychiatric patients (McNair et al., 1992, p. 7). Gibson reports similar values for a non-clinical sample of 60- to 98-year-olds (Gibson, 1997), with the retest reliabilities being higher at a 1-week interval (0.68–0.83) than in McNair et al. (1992). However, the POMS is also used in variants with a 7-step answer scale for each item and an alternative recording period such as “How did you feel today?” (vs. last week including today) e.g., by Gibson (1997).

Concerning the instructions, the requests “How have you felt during the past week including today?” and “How do you feel right now?” are the most commonly used in the POMS. McNair et al. (1992) chose the reference to the past week since they considered this was a long enough period to capture people's typical and persistent emotional reactions to daily life events, yet short enough to assess the acute effects of a treatment. McNair et al. replicated the original structure by using the “right now” instruction in a one factor analysis (McNair et al., 1992). Some researchers have examined the consequences of switching reference times in mood assessment. According to Watson (1988), the structure of positive and negative affect factors emerged regardless of the time frame rated. However, the scope of the reference period will affect an individual's perception of intensity, as well as of seriousness and frequency of the episode under evaluation (Watson, 1988; Schaeffer and Presser, 2003). In the Anglo-Saxon context the scores obtained on the “past week” instruction were higher than the average scores obtained in multiple “right now” assessments (Terry et al., 2005). Recall of mood appeared to be influenced by mood at the time of recall and possible significant events. So, Terry et al., 2005 suggested that the “right now” response time frame should be the method of choice.

Concerning the factorial structure, a conceptual schema underlying the POMS describes the effect of mood states on performance based on the interactions among the POMS' factor scores (Lane and Terry, 2000). This theoretical model emphasized the relationships of depressed mood state with other unpleasant states, particularly tension, and anger. Furthermore, the internal structure of the POMS seems relatively established, with the exception of the Confusion factor (Netz et al., 2005; Morfeld et al., 2007; Bourgeois et al., 2012), which was regarded as a cognitive state (Lane et al., 2007). Moreover, despite the limited range of pleasant mood states covered, for the aforementioned reasons, few adaptations retained the component of Friendliness (Andrade et al., 2010).

Previous research (Watson, 1988; Winkielman et al., 1998; Terry et al., 1999, 2005) has identified additional factors (e.g., diverse mood state descriptors, response formats, or circumstances of assessment) that could affect the understanding and comparability of the mood state responses. Of all the potential modulators, one variable stands out: the circumstances of mood state assessment, i.e., the conditions of time and place under which the mood state response is registered. In this respect, the POMS questionnaire has been administered before and after competition, and outside the competition context (Terry et al., 1999, 2003). Both the type of instruction and the circumstances of administration of the test are important measurement elements. In addition, the invariance of mood scores should be examined and taken into account when interpreting individual and group mood state assessments (Andrade and Rodríguez, 2018).

Besides various translations into Spanish (Andrade et al., 2013), Turkish (Selvi et al., 2011), Arabic (Aroian et al., 2007), Hebru (Netz et al., 2005), and cross-cultural analyses (Yeun and Shin-Park, 2006), various English short forms have been proposed. Cella et al. (1987) developed a version with 11 items as a Total Mood Disturbance without subscales. Shacham (1983) developed a version with 37 items that form six scales. This short version was used in various samples by Curran et al. (1995) and Dilorenzo et al. (1999) with similar good test parameters as for the long form. Baker et al. (2002) were able to confirm the factor structure. In addition, the authors of the original instrument also developed a short form with 30 items that show the same six scales as the long form (McNair et al., 1992).

The German short version by Biehl et al. (1986) consists of 35 items with four scales: dejection/anxiety (14 items), fatigue (seven items), vigor (seven items), and anger (seven items). Similar to the different English versions the German items were answered on a 5- vs. 7-step response and evaluated for different periods “the last 24 h” vs. “the last week including today.” For this German version, satisfactory psychometric properties (internal consistency coefficients of the subscales from *a* = 0.88 to 0.94) with satisfactory factorial validity were reported based on a student sample (Bullinger et al., 1990; Gross, 1991). For the inter-scale-correlations, mean correlation coefficients of *r* = 0.45 (0.44 – 0.65) were determined.

Since the psychometric properties were obtained in a student sample the POMS was implemented in a representative general population sample. Herein, satisfactory internal consistencies between *r* = 0.89 and 0.95 were observed. The factorial structure, however, was only with limited satisfactory (Albani et al., 2005).

Since the factorial structure was only limited satisfactory in a representative large sample, an item selection for a good factorial structure has to be implemented in order to improve the factorial structure. Furthermore, for epidemiological studies short instruments with good psychometric properties are important in order to avoid exhaustion, resistance and boredom. These are important steps in order to be able to use this instrument in large epidemiological longitudinal studies and to get high quality data. In addition, it would be of interest how many items are necessary in order to evaluate the mood construct. Therefore, the aim of our investigation was to empirically identify a short version of the POMS with very good psychometric properties and a good factorial structure of the instrument in a representative population sample.

## Method

The present study was part of a national representative survey of the general population of Germany. Data were collected by an independent institute for opinion and social research (USUMA, Berlin). The criteria for inclusion were a minimum age of 14 and sufficient ability to understand the written German language. After a socio-demographic interview, the participants completed self-report questionnaires regarding physical and psychological symptoms in the presence of (but without any interference from) the interviewer. A random-route sampling procedure with 201 sample points revealed that 3,194 households needed to be contacted for the study. Of these, 3,108 households proved eligible for participation. The selection of the target individuals within the households was carried out according to the Kish selection grid (Kish, 1949). In total, 2,066 individuals took part in the study (participation rate 66%). Subjects with missing data in at least one of the items (*n* = 60) were excluded from the analysis. Thus, the final sample consisted of 2,006 individuals. A detailed description of the sample can be found in Table 1. Mode monthly net income was 1,500€. The procedure of the present study was approved by the ethics committee of the University of Leipzig (043/20-ek). In addition, it adhered to ICH-GCP-guidelines along with the ICC/ESOMAR International Code of Marketing and Social Research Practice. All participants were informed of the study procedures, data collection and anonymization of all personal data. All the participants provided verbal informed consent according to German law, which was documented by the interviewer before starting the survey.

**Table 1:** Sample description with group comparisons for the POMS-16 subscales.

	***n***	**%**	**POMS-D**	**POMS-V**	**POMS-F**	**POMS-A**
Sex			*F*_(1, 2, 064)_ = 21.42, *p* < 0.001, η^2^*_*p*_* = 0.010	*F*_(1, 2, 064)_ = 16.91, *p* < 0.001, η^2^*_*p*_* = 0.008	*F*_(1, 2, 064)_ = 42.13, *p* < 0.001, η^2^*_*p*_* = 0.020	*F*_(1, 2, 064)_ = 0.14, *p* = 0.704, η^2^*_*p*_* = 0.000
Female	1,094	53	3.44 (4.47)	12.13 (5.80)	5.98 (5.17)	6.15 (3.58)
Male	972	47	2.59 (3.83)	13.16 (5.57)	4.57 (4.62)	6.38 (3.47)
Age (in years; *M* = 48.83; *SD* = 18.11)			*F*_(5, 2, 060)_ = 4.73, *p* < 0.001, η^2^*_*p*_* = 0.011	*F*_(5, 2, 060)_ = 34.51, *p* < 0.001, η^2^*_*p*_* = 0.077	*F*_(5, 2, 060)_ = 16.31, *p* < 0.001, η^2^*_*p*_* = 0.038	*F*_(5, 2, 060)_ = 4.11, *p* = 0.001, η^2^*_*p*_* = 0.010
14–29	374	18.1	2.43 (3.76)	14.18 (6.01)	4.05 (4.70)	6.57 (3.64)
30–39	316	15.3	2.81 (4.03)	13.91 (5.45)	5.37 (4.94)	7.10 (3.54)
40–49	356	17.2	2.92 (4.20)	13.46 (5.48)	5.18 (4.89)	6.64 (3.40)
50–59	328	15.9	3.29 (4.14)	12.73 (5.27)	5.09 (4.40)	6.27 (3.21)
60–69	391	18.9	3.05 (4.11)	11.68 (5.37)	5.20 (4.62)	5.91 (3.49)
≥70	301	14.6	3.92 (4.87)	9.38 (5.27)	7.41 (5.75)	4.96 (3.53)
Education			*F*_(2, 2, 063)_ = 13.26, *p* < 0.001, η^2^*_*p*_* = 0.013	*F*_(2, 2, 063)_ = 39.80, *p* < 0.001, η^2^*_*p*_* = 0.037	*F*_(2, 2, 063)_ = 13.24, *p* < 0.001, η^2^*_*p*_* = 0.013	*F*_(2, 2, 063)_ = 4.37, *p* = 0.013, η^2^*_*p*_* = 0.004
≤9 years	997	48.3	3.51 (4.54)	11.48 (5.69)	5.84 (5.13)	5.87 (3.50)
10 years	803	38.9	2.74 (3.90)	13.75 (5.53)	4.64 (4.58)	6.70 (3.42)
≥11 years	266	12.9	2.24 (3.48)	13.45 (5.53)	5.43 (5.21)	6.39 (3.81)
Family			*F*_(5, 2, 060)_ = 4.20, *p* = 0.001, η^2^*_*p*_* = 0.010	*F*_(5, 2, 060)_ = 27.01, *p* < 0.001, η^2^*_*p*_* = 0.062	*F*_(5, 2, 060)_ = 11.67, *p* < 0.001, η^2^*_*p*_* = 0.028	*F*_(5, 2, 060)_ = 4.37, *p* = 0.001, η^2^*_*p*_* = 0.010
Married	1,024	49.6	2.91 (4.14)	13.01 (5.33)	5.09 (4.87)	6.44 (3.41)
Unmarried, living with partner	89	4.3	2.07 (3.04)	14.26 (5.18)	5.24 (4.46)	6.82 (3.41)
Unmarried	419	20.3	2.77 (4.05)	13.85 (5.96)	4.45 (4.86)	6.60 (3.66)
Separated	24	1.2	2.96 (3.60)	11.44 (6.08)	5.28 (4.45)	6.64 (3.03)
Divorced	221	10.7	3.73 (4.51)	12.15 (6.08)	5.60 (5.01)	6.48 (3.70)
Widowed	288	13.9	3.71 (4.58)	9.37 (5.26)	7.20 (5.17)	4.74 (3.33)
Employment			*F*_(3, 2, 062)_ = 14.90, *p* < 0.001, η^2^*_*p*_* = 0.021	*F*_(3, 2, 062)_ = 52.16, *p* < 0.001, η^2^*_*p*_* = 0.071	*F*_(3, 2, 062)_ = 12.82, *p* < 0.001, η^2^*_*p*_* = 0.018	*F*_(3, 2, 062)_ = 5.65, *p* = 0.001, η^2^*_*p*_* = 0.008
Working	902	43.7	2.41 (3.55)	14.01 (5.28)	4.75 (4.59)	6.65 (3.36)
Not working	351	17.0	3.99 (4.77)	12.42 (5.98)	5.46 (4.96)	6.68 (3.61)
Retired	649	31.4	3.43 (4.54)	10.53 (5.38)	6.23 (5.31)	5.42 (3.52)
In training	164	7.9	2.96 (4.26)	13.60 (6.17)	4.55 (5.01)	6.51 (3.71)

*D, Dejection; V, Vigor; F, Fatigue; A, Anger*.

### Instruments

In the present study the German short version of the Profile of Mood States (POMS; McNair et al., 1981) by Biehl et al. (1986) was implemented. The German short version consists of 35 items with four scales: dejection/anxiety (14 items), fatigue (seven items), vigor (seven items), and anger (seven items). Similar to the different English versions the German items were answered on a 5- vs. 7-step response and evaluated for “the last 24 h” (McNair et al., 1981).

### Statistical Analysis

All analyses were conducted in R, using the packages *EFAutilities, lavaan, moments, multilevel, semTools*, and *stuart* (Rosseel, 2012; Komsta and Novomestky, 2015; Bliese, 2016; Dinno, 2018; Jorgensen et al., 2019; Schultze, 2019; Zhang et al., 2019). Initially, we randomly split our total sample (*n* = 2,006) into an exploratory (*n* = 1,029) and a confirmatory (*n* = 977) one. For the shortening of the scale in the exploratory analyses, we first examined the item descriptive statistics—in addition to running parallel analysis (Horn, 1965). For the parallel analysis, we utilized the full correlation matrix. We report both, a factor analytical and a principal component based approach with unweighted least squares estimation. However, the primary technique we employed to shorten the scale is based on model comparisons of potential item subsets (Schultze, 2019). We decided to look for solutions with four items per factor to balance the contrasting demands of scale brevity and reliability/validity. On the one hand, the aim of this study was to create the shortest possible assessment of mood. On the other hand, excessively short scales can struggle with capturing the entire width of a given construct reliably. That is first, content validity may be compromised, and second, composite reliability suffers exponentially greater losses for each item removed the fewer items remain. In addition, we constrained the algorithm to prefer solutions that are strongly invariant across participant sex. The *stuart* algorithm uses ant-colony optimization as a search strategy in constructing and testing subsets for the given model parameters. This avoids the necessity of testing all of the many possible combinations.

To affirm the model configuration gained in the exploratory analyses we then computed a confirmatory factor analysis (CFA) using robust maximum likelihood estimation (Yuan and Bentler, 2000). We evaluated model fit using the χ^2^-test and the common descriptive fit indices: the Comparative Fit Index (*CFI*), the Tucker-Lewis Index (*TLI*), the Root Mean Square Error of Approximation (*RMSEA*), and the Standardized Root Mean Square Residual (*SRMR*). According to the typical recommendations, *CFI* and *TLI* should be at least 0.95 and *RMSEA* and *SRMR* should be no >0.08 or preferably 0.05 (Hu and Bentler, 1999; Schermelleh-Engel et al., 2003).

We report omega as a measure of internal consistency. Since the multiple factors in our model are only moderately correlated and no second-order or general construct can be assumed, we utilized McDonald's (1999) basic formula. In addition, we tested the emerging model for measurement invariance across age groups and participant sex. To this end, we employed the common procedure described by Meredith (1993) of successively constraining factor loadings, item intercepts, and item residual variances. As per recommendations found in the literature (Cheung and Rensvold, 2002; Chen, 2007), we utilized between-model cutoffs of 0.010 for *CFI* and 0.015 for *RMSEA*. Where applicable, we calculated effect size estimates for group-specific parameter differences. To this end, we refered to the formulas supplied by Pornprasertmanit (2019), but corrected them according to Cohen (1988; see Supplemental Material).

## Results

### Exploratory Analyses

The parallel analysis using principal components evinced evidence for a four-factorial solution, as the empirical eigenvalues (15.55, 3.73, 1.70, 1.44, 0.97,.) were larger than the randomly generated ones (including a 99% confidence interval: 1.36, 1.32, 1.28, 1.25, 1.23,.) up until the fourth component. On the other hand the parallel analysis using factor analysis indicated five factors with empirical eigenvalues (15.05, 2.97, 1.13, 0.84, 0.47, 0.22,.) exceeding the simulated ones for the first five instances (including a 99% confidence interval: 0.39, 0.33, 0.29, 0.26, 0.24, 0.22,.). Because the POMS-35 was conceptualized as a four-factor instrument, we decided on utilizing four factors moving forward.

We then investigated the loading patterns of the 35 initial POMS items (Table 2), removing those items from consideration that either had no loadings of 0.50 on at least one factor, and those items that exhibited substantial loadings (λ ≥ 0.25) on more than one factor (3, 6, 7, 10, 11, 12, 19, 21, 25). These cutoffs were based on the common recommendation of excluding items with unclear loading patterns. Since *r* = 0.50 is generally regarded as the threshold for a high association, this was the inclusion criterion on the item's main factor. To our knowledge there are no clearly established rules for the acceptable magnitude of cross-loadings. For this reason we selected half of the previous value, *r* = 0.25, as the maximum permissible value for cross-loadings. Next, we explored the item response distributions, and removed items with absolute skewness >2 and/or absolute excessive kurtosis >4 (19, 24, 26, 32, 33), as such values indicate non-normal distributions (Kim, 2013). We then checked the corrected item-total correlations (*r*_*it*_) for the remaining items based on the scales that were suggested by the loading patterns in Table 2. This led to no further exclusions based on the *r*_*it*_ ≥ 0.50 criterion. Finally, we removed Items 15 (“spiteful”) and 20 (“cheerful”) based on higher intercorrelations and theoretical considerations (high convergence).

**Table 2:** Item descriptive statistics and exploratory factor analysis of the POMS-35.

	***M***	***SD***	**γ1**	**γ2**	**λ(F1)**	**λ(F2)**	**λ(F3)**	**λ(F4)**	**λ(CFA)**	**λ(F1_**ESEM**_)**	**λ(F2 _**ESEM**_)**	**λ(F3 _**ESEM**_)**	**λ(F4 _**ESEM**_)**	**ω**
Item 1^A^	0.78	1.22	1.61	5.00	−0.01	−0.05	−0.08	**0.74**	**0.72**	**0.44**	−0.07	0.15	**0.63**	
Item 2^F^	1.30	1.45	1.02	3.27	−0.03	−0.06	**0.74**	0.08	**0.81**	0.13	−0.13	**0.71**	0.12	
Item 3	0.85	1.29	1.56	4.75	**0.49**	−0.13	0.20	0.12						
Item 4^V^	3.10	1.58	−0.30	2.42	0.03	**0.70**	−0.05	0.02	**0.67**	−0.05	**0.59**	−0.06	0.12	
Item 5^D^	0.90	1.18	1.37	4.43	**0.51**	−0.02	0.22	0.11	**0.70**	**0.63**	−0.01	0.13	0.08	
Item 6	1.11	1.34	1.16	3.60	**0.30**	−0.16	**0.45**	0.08						
Item 7	0.90	1.31	1.49	4.54	**0.46**	−0.12	0.23	0.15						
Item 8	3.68	1.54	−0.63	2.86	−0.09	**0.72**	−0.06	0.03						
Item 9^A^	1.20	1.32	1.01	3.37	−0.05	0.05	0.17	**0.74**	**0.78**	**0.45**	0.01	0.27	**0.62**	
Item 10	0.79	1.22	1.60	4.84	**0.33**	−0.04	0.18	0.40						
Item 11	0.92	1.32	1.43	4.18	**0.47**	−0.09	0.29	0.18						
Item 12	2.81	1.75	−0.18	2.05	−0.08	**0.58**	0.01	**0.30**						
Item 13	0.65	1.13	1.97	6.64	**0.59**	0.00	0.14	0.09						
Item 14^D^	0.80	1.31	1.70	5.18	**0.60**	−0.15	0.08	0.10	**0.82**	**0.86**	−0.07	−0.05	0.01	
Item 15	0.86	1.26	1.48	4.68	0.01	−0.03	0.22	**0.63**						
Item 16^F^	1.69	1.48	0.66	2.80	−0.13	−0.06	**0.79**	0.05	**0.76**	0.06	−0.08	**0.72**	0.07	
Item 17^A^	1.06	1.32	1.18	3.68	0.00	−0.01	0.14	**0.73**	**0.85**	**0.56**	−0.01	0.25	**0.59**	
Item 18^D^	0.81	1.28	1.73	5.50	**0.58**	−0.12	0.18	0.19	**0.89**	0.87	−0.03	0.05	0.10	
Item 19	0.41	0.86	2.37	8.60	0.29	0.00	−0.08	**0.30**						
Item 20	3.54	1.40	−0.44	2.92	−0.02	**0.72**	0.01	−0.11						
Item 21	0.65	1.14	1.97	6.61	**0.42**	−0.09	−0.06	**0.46**						
Item 22^F^	1.44	1.44	0.83	2.92	−0.05	−0.07	**0.83**	0.06	**0.86**	0.09	−0.03	**0.82**	0.02	
Item 23^D^	0.67	1.18	1.90	6.09	**0.58**	−0.09	0.17	0.14	**0.80**	**0.63**	−0.06	0.25	0.07	
Item 24	0.61	1.18	2.07	6.59	**0.65**	−0.08	0.08	0.20						
Item 25	0.85	1.27	1.56	4.79	**0.30**	−0.08	**0.47**	0.07						
Item 26	0.59	1.14	2.14	7.12	**0.64**	−0.07	0.23	0.03						
Item 27^F^	1.05	1.35	1.25	3.76	0.16	−0.05	**0.72**	0.01	**0.82**	0.25	−0.06	**0.64**	0	
Item 28^V^	3.46	1.57	−0.54	2.70	0.04	**0.76**	−0.05	−0.07	**0.76**	−0.04	**0.71**	−0.06	0.01	
Item 29^A^	0.92	1.26	1.41	4.44	0.07	0.01	−0.04	**0.76**	**0.81**	**0.53**	0.03	0.18	**0.65**	
Item 30^V^	2.99	1.71	−0.32	2.17	0.07	**0.79**	−0.05	0.00	**0.87**	0.01	**0.87**	−0.03	0	
Item 31	0.87	1.15	1.34	4.22	0.24	−0.06	0.05	**0.55**						
Item 32	0.42	0.91	2.49	9.15	**0.71**	−0.04	−0.02	0.13						
Item 33	0.48	1.00	2.38	8.46	**0.55**	−0.02	−0.01	0.22						
Item 34^V^	3.34	1.70	−0.46	2.38	−0.01	**0.77**	−0.01	0.02	**0.77**	−0.12	**0.76**	0.05	−0.03	
Item 35	0.98	1.33	1.39	4.26	0.27	−0.06	**0.60**	0.04						
Dejection	3.10	4.20	1.64	5.38										0.88
Vigor	12.89	5.47	−0.27	2.47										0.86
Fatigue	5.40	4.94	1.03	3.78										0.88
Anger	6.38	3.42	0.69	3.74										0.86

*M, Mean; SD, Standard Deviation; γ1, Skewness; γ2, Kurtosis; λ(Fx), EFA loadings on factor x; λ(CFA), CFA loadings on respective factors; λ(Fx_ESEM_), ESEM loadings on factor x; ω, McDonald's omega reliability coefficient; ^D^, Dejection items; ^V^, Vigor items; ^F^, Fatigue items; ^A^, Anger items. Bolded values indicate significant factor loadings, λ ≥ 0.30*.

The remaining 20 items were then entered into the exploratory *stuart* algorithm in order to look for the optimal four-factorial solution with four items per factor. Since there were only 625 possible solutions, we used the bruteforce algorithm—meaning that all solutions were tested. Among those, the items marked in Table 2 were selected. This model evinced good fit, χ^2^(220) = 360.445, *p* < 0.001, *CFI* = 0.979, *TLI* = 0.977, *RMSEA* = 0.042, *SRMR* = 0.039, and very good reliability for the subscales ω_Dejection_ = 0.882 [0.863, 0.901], ω_Vigor_ = 0.855 [0.836, 0.873], ω_Fatigue_ = 0.883 [0.867, 0.900], ω_Anger_ = 0.862 [0.843, 0.882]. Because of its vastly superior factor loading we replaced Item 31 (“bad tempered”) with Item 1 (“angry”).

### Factorial Validity

We then computed a CFA with the above-mentioned 16 items in a correlated factors model with four latent constructs. This model exhibited good fit, χ^2^(98) = 315.394, *p* < 0.001, *CFI* = 0.967, *TLI* = 0.960, *RMSEA* = 0.056, *SRMR* = 0.037, and again very good reliability for the subscales (see Table 2). All standardized factor loadings were equal to or >0.671. Following the advice of a reviewer, we also tested an exploratory structural equation model (ESEM; Asparouhov and Muthén, 2009) for the same input data. This analysis presented further evidence for the psychometric quality of the selected items. That is, the items separated nicely onto the four factors for the most part. Some cross-loadings between the anger and dejection items became appearant. As expected, model fit was improved over the CFA, χ^2^(56) = 123.51, *p* < 0.001, *CFI* = 0.988, *TLI* = 0.975, *RMSEA* = 0.035, *SRMR* = 0.014.

We then compared the 16-item version of the POMS to the full 35-item version. All four subscales of the short scale correlated very highly (*r* > 0.95) with the original. To free the associations from autocorrelations, we then removed those items that were selected for the POMS-16 from the original subscales and recalculated the correlations. The associations were attenuated but still high: *r*_Dejection_= 0.90, *r*_Vigor_= 0.79, *r*_Fatigue_ = 0.80, *r*_Anger_ = 0.79. Additionally, we compared the subscale- and factor-intercorrelations (see Table 3). Here it became obvious that correlations for the shortened POMS-16 are slightly diminished when compared to the POMS-35. Although this comparison yielded significant differences in six of the 12 cases (given α = 0.05, no correction for multiple testing), the average standardized difference between the correlation coefficients was very small $\bar{q}$ = 0.071. This can be considered evidence that the shortened POMS-16 still accurately captures the original constructs and retains their associations.

**Table 3:** Observed and latent correlations for the POMS-35 and−16 subscales.

	**Dejection**	**Vigor**	**Fatigue**	**Anger**
	**POMS-35**			
Dejection	1	−0.382	0.733	0.754
Vigor	−0.393	1	−0.461	−0.167
Fatigue	0.780	−0.496	1	0.612
Anger	0.820	−0.174	0.668	1
	**POMS-16**			
Dejection	1	−0.373	0.621	0.617
Vigor	−0.375	1	−0.435	−0.128
Fatigue	0.694	−0.465	1	0.471
Anger	0.712	−0.111	0.525	1

*Observed correlations are above the diagonal, latent correlations below the diagonal*.

Next, we tested this model for measurement invariance across participant sex and age groups. Since we had used the exploratory subsample to look for a model configuration that is strongly invariant across sex, we used only the confirmatory subsample to test invariance across sex. For the age groups, we utilized the full sample. We report the results of the step-wise test process in Table 4. The model is strictly invariant across age groups. In terms of participant sex, there is evidence for strong invariance. In addition, we demonstrated partial strict invariance by freeing the intercept of Item 23 (“gloomy”) to vary between groups. The group-specific residuals of this item differed by a moderate effect size, *h* = 0.442.

**Table 4:** Test of measurement invariance across age and sex for the POMS-16.

**Model**	**χ^2^ (*df*)**	**Δχ^2^**	**Δ*df***	***p***	***CFI***	**Δ*CFI***	***RMSEA***	**Δ*RMSEA***
**Sex**								
Configural invariance	422.060 (196)				0.966		0.057	
Metric invariance	439.287 (208)	17.227	12	0.141	0.965	0.001	0.056	0.001
Scalar invariance	468.463 (220)	29.176	12	0.004	0.963	0.002	0.056	0.000
Strict invariance	543.819 (236)	75.356	16	<0.001	0.952	0.011	0.062	0.006
Partial strict invariance^a^	515.318 (235)	46.855	15	<0.001	0.956	0.007	0.059	0.003
**Age**								
Configural invariance	1034.999 (588)				0.967		0.055	
Metric invariance	1134.212 (648)	99.213	60	0.001	0.964	0.003	0.055	0.000
Scalar invariance	1286.263 (708)	152.051	60	<0.001	0.958	0.006	0.057	0.002
Strict invariance	1435.901 (788)	149.638	80	<0.001	0.950	0.008	0.059	0.002

a *The residual of Item 23 (“gloomy”) was freed to vary between groups*.

### Socioemographic Differences and Norm Values

Before computing norm values, we examined how the various sociodemographic variables in the data set influenced the POMS-16 scores. It should be noted that most comparisons were (highly) significant, simply due to the large sample size. In order to gain an understanding of the practical relevance of these effects we consulted the effesct size measure η^2^. For η^2^, 0.01 indicates small effects, 0.06 indicates moderate effects, and 0.14 indicates large effects (Cohen, 1988). For the Dejection subscale, all effects should be categorized as small, with employment status being the strongest factor. With regard to the Vigor subscale, we found moderate effects for age, employment status, and family status. Education had a small to moderate effect on Vigor. Age and family status also significantly affected the Fatigue subscale, with small to moderate effects. In terms of the Anger subscale, none of the predictors had more than a negligibly small influence on the scale score. Monthly net income had small negatives association with Dejection (*r* = −0.108, *p* < 0.001) and Fatigue (*r* = −0.062, *p* = 0.006), a small positive association with Vigor (*r* = 0.122, *p* < 0.001), and a near-zero correlation with Anger (*r* = 0.031, *p* = 0.156).

Subsequently, we calculated norm values for the newly constructed POMS-16 short form in Table 5. Even though, all sociodemographic variables under study had significant effects on one or more of the subscale scores, it was not feasible in terms of space to account for all sources of influence. We thus elected to stratify the norm data by participant sex and age groups. Based on these values, individual scores can be compared to the distributions found in the German general population.

**Table 5:** Percentile rank normative values for the POMS-16, stratified by sex and age.

	**Females**																							
**Age in years**	**14–29 (*****n*** **=** **179)**				**30–39 (*****n*** **=** **174)**				**40–49 (*****n*** **=** **174)**				**50–59 (*****n*** **=** **172)**				**60–69 (*****n*** **=** **193)**				**≥70 (*****n*** **=** **202)**			
**Sum score**	**D**	**V**	**F**	**A**	**D**	**V**	**F**	**A**	**D**	**V**	**F**	**A**	**D**	**V**	**F**	**A**	**D**	**V**	**F**	**A**	**D**	**V**	**F**	**A**
0	42	0	26	2	39	1	15	2	40	1	17	3	34	1	16	4	37	2	13	5	28	7	5	8
1	60	1	37	3	51	2	21	3	51	2	23	5	46	1	22	5	51	4	18	9	41	10	9	12
2	65	3	47	8	59	5	28	4	56	5	32	8	54	2	31	9	56	6	28	14	47	13	17	24
3	74	5	55	12	66	5	36	11	62	8	40	12	61	6	37	18	65	11	37	21	57	15	24	37
4	78	6	63	24	74	6	45	17	67	9	52	24	69	9	46	29	69	14	44	40	61	20	31	51
5	84	7	66	36	78	9	57	37	74	11	58	36	73	12	53	48	73	17	51	53	66	24	36	65
6	85	14	71	55	83	12	62	51	77	14	65	53	78	13	62	61	77	19	59	67	72	30	45	76
7	86	15	75	65	84	15	65	64	80	18	69	66	83	18	68	71	81	26	64	72	76	37	50	80
8	89	18	79	75	90	19	74	70	84	21	73	70	88	22	74	78	85	32	72	84	80	44	58	84
9	91	24	82	80	92	22	80	73	85	26	74	76	91	25	79	84	88	38	80	88	84	48	65	88
10	93	27	88	86	93	26	85	82	89	31	81	84	93	32	85	89	90	42	83	92	88	56	69	90
11	94	30	89	89	95	34	88	88	92	36	82	88	95	42	88	92	93	48	86	93	89	64	77	92
12	95	35	92	92	95	36	90	92	94	44	86	93	96	47	91	95	96	58	88	95	92	72	83	96
13	97	40	94	95	97	40	91	98	95	48	88	96	97	54	95	99	97	64	92	99	93	77	88	98
14	97	45	95	97	97	46	95	99	96	52	92	98	98	60	95	99	97	70	94	99	96	83	90	99
15	98	52	96	98	98	59	96	99	98	61	92	99	99	68	97	99	98	75	95	99	97	88	92	99
16	99	62	97	99	98	67	97	99	98	66	95	99	99	76	99	99	99	83	96	100	97	93	92	99
17	99	68	97	99	99	74	97	99	98	72	98	100	99	81	99	100	99	87	96		98	94	93	100
18	100	76	98	99	99	83	97	99	99	78	99		99	87	99		99	91	98		98	98	94	
19		81	98	100	99	87	98	99	100	87	99		99	94	99		99	93	99		99	99	96	
20		89	98		99	94	98	100		92	100		99	95	99		99	98	100		99	100	97	
21		95	99		100	97	99			94			99	97	99		99	99			100		97	
22		97	99			98	99			98			99	99	99		100	99					98	
23		98	100			99	99			98			100	99	100			99					99	
24		100				100	100			100				100				100					100	
	**Males**																							
**Age in years**	**14–29 (*****n*** **=** **195)**				**30–39 (*****n*** **=** **142)**				**40–49 (*****n*** **=** **182)**				**50–59 (*****n*** **=** **156)**				**60–69 (*****n*** **=** **198)**				**≥70 (*****n*** **=** **99)**			
**Sum Score**	**D**	**V**	**F**	**A**	**D**	**V**	**F**	**A**	**D**	**V**	**F**	**A**	**D**	**V**	**F**	**A**	**D**	**V**	**F**	**A**	**D**	**V**	**F**	**A**
0	45	3	33	4	43	0	20	2	49	1	22	2	39	1	19	1	43	2	19	3	48	4	16	9
1	61	3	44	8	60	1	30	4	59	2	31	3	50	3	27	2	58	3	30	5	59	7	21	14
2	70	4	51	9	69	2	37	5	72	4	43	6	59	5	36	6	63	4	42	11	62	8	29	17
3	75	5	59	15	77	4	48	8	78	4	51	11	66	5	46	12	72	4	54	20	70	13	38	39
4	83	6	66	26	82	6	58	22	82	6	60	27	74	8	57	29	78	6	61	32	74	17	48	51
5	85	9	72	41	84	8	65	35	85	8	64	44	77	9	62	47	80	10	65	45	77	22	53	62
6	89	11	77	58	87	10	72	47	86	11	71	56	81	10	70	58	83	11	69	59	81	31	56	68
7	90	13	83	66	89	12	77	57	88	14	75	67	86	14	79	69	87	15	76	71	82	33	64	81
8	93	15	86	74	91	15	83	69	91	16	78	75	90	19	83	74	90	21	78	79	86	36	69	84
9	94	21	89	82	93	18	86	77	92	20	82	81	91	25	88	83	92	30	86	82	90	39	74	89
10	95	25	92	89	94	21	91	84	93	25	87	89	92	30	93	89	94	36	90	88	91	49	77	94
11	97	28	96	91	95	25	92	87	94	30	90	92	92	36	95	95	96	43	92	91	94	56	81	96
12	98	36	96	95	97	32	94	91	98	36	93	96	95	42	95	97	97	51	95	94	95	67	83	99
13	98	42	96	96	97	36	94	94	99	43	95	98	97	48	96	98	98	59	96	95	96	74	85	99
14	98	44	98	97	97	41	94	96	99	53	97	98	98	55	97	99	98	64	97	96	96	81	86	100
15	99	49	98	99	98	48	94	98	99	61	98	98	99	63	98	99	99	73	98	97	96	86	86	
16	99	57	99	99	98	58	96	98	100	70	98	98	99	74	99	99	100	83	100	99	99	93	89	
17	99	66	99	99	98	69	97	99		77	99	100	99	79	99	100		87		99	100	94	92	
18	99	72	99	99	100	76	97	99		81	100		100	87	99			87		99		96	94	
19	99	78	99	99		82	98	99		89				90	100			91		100		99	95	
20	100	84	99	99		92	98	100		93				95				94				99	96	
21		90	99	99		97	99			95				97				96				99	99	
22		90	100	99		99	100			96				99				98				100	100	
23		92		99		99				98				99				99						
24		100		100		100				100				100				100						

*D, Dejection; V, Vigor; F, Fatigue; A, Anger*.

## Discussion

The questionnaire “Profile of Mood States” (POMS; McNair et al., 1992) is a widely used questionnaire in the clinical field. The German version was developed based on several student samples and tested in one representative population sample. The results of the representative sample showed that the factorial structure was only limited. Due to the limited factorial structure, an item selection for a good factorial structure has to be implemented in order to improve the factorial structure. For epidemiological studies short instruments with good psychometric properties are important, therefore, it would be of interest how many items are necessary in order to evaluate the mood construct with good factorial structure. Therefore, in the present study the German version of the POMS was shortened in order to get the best factorial structure. Herefore, a representative population sample was the basis.

Based on an exploratory factor analysis and an exploaratory stuart algorithm a set of items were identified for a good reliabilty and factoriral structure. When this model was tested by a confirmatory factoranalysis this model exhibited good fit, and again very good reliability for the subscales between 0.86 to 0.91. This version with 16 items with four items for each of the four scales is strictly invariant across age groups. In terms of participant sex, there is evidence for strong invariance and partial strict invariance.

For the English speaking POMS exclusively the version by Cella et al. (1987) has a similar number of items (11 items). However, this version only provides a Total Mood Disturbance without subscales. Therefore, this German version with 16 items is the shortest version of the POMS available. It is highly correlated with the 35-item version, which is evidence of concurrent validity. Furthermore, its reliability is remarkably high, considering the brevity of its subscales.

Besides the strength of a large sample size, namely the wide range of ages and the representativeness for the general population, as a limitation, the results cannot necessarily be applied to samples with changed mood. In turn, the POMS needs to be applied to different groups with different moods as well as to clinical samples in order to further replicate or reprobate the factorial structure. Similarly, the inspection of validity needs to be expanded by calculating correlations with other related measures. In addition, there are already several short versions of the original English questionnaire. Therefore, a simple translation one of these could have been an alternative approach and therefore, a lack of novelty. However, an item reduction based on the German long version leads to better psychometric properties and account better for possible cultural differences. Finally, we did not account for response biases such as acquiescence, careless responding and others. Previous research has shown the impact of such processes and how to deal with them (Podsakoff et al., 2003; Maydeu-Olivares and Coffman, 2006; Schmalbach et al., 2020). Also, we did not exclude outliers in order to retain the representativeness of our sample. Future research should thus take these into account to yield a more differentiated view of mood assessment, which may be particularly susceptible to such ephemeral influences.

## Data Availability Statement

The data analyzed in this study is subject to the following licenses/restrictions: the datasets that were analyzed for this article are available from the corresponding author upon reasonable request. Requests to access these datasets should be directed to bjarne.schmalbach@gmail.com.

## Ethics Statement

The studies involving human participants were reviewed and approved by University of Leipzig Medical Faculty Ethics Committee Käthe-Kollwitz-Str. 82 04109 Leipzig. Written informed consent to participate in this study was provided by the participants' legal guardian/next of kin.

## Author Contributions

All authors listed have made a substantial, direct and intellectual contribution to the work, and approved it for publication.

## Conflict of Interest

The authors declare that the research was conducted in the absence of any commercial or financial relationships that could be construed as a potential conflict of interest.
