# Supplementary material for: Revised Short Screening Version of the Profile of Mood States (POMS) From the German General Population
Source: Front Psychol. 2021 May 31;12:631668. doi: 10.3389/fpsyg.2021.631668 (PMC8200627; doi:10.3389/fpsyg.2021.631668)
Supplement: Supplementary file 1 [file Table_1.DOCX]

Supplemental Material

Pornprasertmanit (2019) presents the following formula for calculating the effect size of the difference between two item residual variances:

*h* = $\sin^{-1} \sqrt{\frac{\theta_{Group A}}{\sigma_{P}}}$ - $\sin^{-1} \sqrt{\frac{\theta_{Group B}}{\sigma_{P}}}$ (1).

There are two issues with this conceptualization. First according to Cohen (1988), the correct transformation for comparing proportions is the arcsine of the square root of the proportion, multiplied by two. By leaving out this factor, the resulting effect size estimate will be exactly half of the correct value. Second, the denominator needs to be the pooled variance, not the pooled standard deviation. Per definition, the value of a proportion is between 0 and 1. Since the residual θ is typically given in the form of a variance the total observed variation in the denominator needs to be in the same metric, σ². This is particularly important with σ > 1: The larger the variance, σ², the smaller the standard deviation, σ, will be. As a result, the value of the fraction can be greater than 1, when employing σ_P_, which is problematic for the arcsine transformation.

The correct formula, thus needs to be put as:

*h* = $2* \sin^{-1} \sqrt{\frac{\theta_{Group A}}{{\sigma^{2}}_{P}}}$ - $2* \sin^{-1} \sqrt{\frac{\theta_{Group B}}{{\sigma^{2}}_{P}}}$ (2),

or in its simplified form as

*h* = $2*{(sin}^{-1} \sqrt{\frac{\theta_{Group A}}{{\sigma^{2}}_{P}}}$ - $\sin^{-1} \sqrt{\frac{\theta_{Group B}}{{\sigma^{2}}_{P}}})$ (3).
